# Supplementary material for: The spleen bacteriome of wild rodents and shrews from Marigat, Baringo County, Kenya
Source: PeerJ. 2021 Sep 2;9:e12067. doi: 10.7717/peerj.12067 (PMC8418798; doi:10.7717/peerj.12067)
Supplement: Supplemental Information 1 — Note: Under the primer column, the letters “L” and “H” identify the light (L) and heavy (H) chains respectively and the number gives the position of the 3′ base of the primer. # = primers used for nested PCR. [file peerj-09-12067-s001.docx]

**Table S1. Primers used for amplification and sequencing of *Cyt b* and 16S rRNA genes.** *Note:* Under the primer column, the letters “L” and “H” identify the light (L) and heavy (H) chains respectively and the number gives the position of the 3′ base of the primer. # = primers used for nested PCR.

| Gene Target | Primer | Primer sequence (5’ - 3’) | Fragment size | Source |
| --- | --- | --- | --- | --- |
| *Cytb* | L14723 | 5’-CCAATGACATGAAAAATCATCGTT-3 | 1140bp | (Nicolas et al., 2012) |
|  | H15915 | TCTCCATTTCTGGTTTACAAGAC |  |  |
|  | ^#^L14749 | ACGAAACAGGCTCTAATAA |  |  |
|  | ^#^H14896 | TAGTTGTCGGGGTCTCCTA |  |  |
| 16S rRNA | Forward primer | TCGTCGGCAGCGTCAGATGTGTATAAGAGACAGCCTACGGGNGGCWGCAG | 460bp | (Klindworth et al., 2013) |
|  | Reverse primer | GTCTCGTGGGCTCGGAGATGTGTATAAGAGACAGGACTACHVGGGTATCTAATCC |  |  |
