# Supplementary material for: The spleen bacteriome of wild rodents and shrews from Marigat, Baringo County, Kenya
Source: PeerJ. 2021 Sep 2;9:e12067. doi: 10.7717/peerj.12067 (PMC8418798; doi:10.7717/peerj.12067)
Supplement: Supplemental Information 2 [file peerj-09-12067-s002.docx]

**Table S2. Number of reads in non-template control sample that was used to track contaminant OTUs**

| OTU_ID | Total reads | Taxonomy |
| --- | --- | --- |
| Otu000036 | 66 | *Bacteria;Proteobacteria;Proteobacteria_unclassified;Proteobacteria_unclassified;Proteobacteria_unclassified;NA* |
| Otu000039 | 84 | *Bacteria;Actinobacteria;Actinobacteria;Actinomycetales;Dermacoccaceae;Dermacoccus* |
| Otu000048 | 44 | *Bacteria;Bacteroidetes;Sphingobacteria;Sphingobacteriales;Cytophagaceae;Hymenobacter* |
| Otu000054 | 54 | *Bacteria;Proteobacteria;Alphaproteobacteria;Caulobacterales;Caulobacteraceae;Brevundimonas* |
| Otu000070 | 69 | *Bacteria;Proteobacteria;Betaproteobacteria;Burkholderiales;Comamonadaceae;Variovorax* |
| Otu000086 | 54 | *Bacteria;Proteobacteria;Alphaproteobacteria;Caulobacterales;Caulobacteraceae;Brevundimonas* |
| Otu000087 | 140 | *Bacteria;Actinobacteria;Actinobacteria;Actinomycetales;Geodermatophilaceae;Blastococcus* |
